# Virtual Screening Using Pharmacophore Models Retrieved from Molecular Dynamic Simulations

**DOI:** 10.3390/ijms20235834

**Published:** 2019-11-20

**Authors:** Pavel Polishchuk, Alina Kutlushina, Dayana Bashirova, Olena Mokshyna, Timur Madzhidov

**Affiliations:** 1 Institute of Molecular and Translational Medicine, Faculty of Medicine and Dentistry, Palacky University and University Hospital in Olomouc, Hnevotinska 5, 77900 Olomouc, Czech Republic; alina.kutlushina@upol.cz (A.K.); olena.mokshyna@upol.cz (O.M.); 2 A.M. Butlerov Institute of Chemistry, Kazan Federal University, Kremlyovskaya Str. 18, 420008 Kazan, Russia; dayana.bashirova@yandex.ru (D.B.); timur.madzhidov@kpfu.ru (T.M.)

**Keywords:** pharmacophore, molecular dynamics, virtual screening

## Abstract

Pharmacophore models are widely used for the identification of promising primary hits in compound large libraries. Recent studies have demonstrated that pharmacophores retrieved from protein-ligand molecular dynamic trajectories outperform pharmacophores retrieved from a single crystal complex structure. However, the number of retrieved pharmacophores can be enormous, thus, making it computationally inefficient to use all of them for virtual screening. In this study, we proposed selection of distinct representative pharmacophores by the removal of pharmacophores with identical three-dimensional (3D) pharmacophore hashes. We also proposed a new conformer coverage approach in order to rank compounds using all representative pharmacophores. Our results for four cyclin-dependent kinase 2 (CDK2) complexes with different ligands demonstrated that the proposed selection and ranking approaches outperformed the previously described common hits approach. We also demonstrated that ranking, based on averaged predicted scores obtained from different complexes, can outperform ranking based on scores from an individual complex. All developments were implemented in open-source software pharmd.

## 1. Introduction

Pharmacophore models are widely used in the early stages of drug development to identify potential hits in large datasets. These models encode spatial arrangements of features which are important for protein–ligand interactions and can be derived from available three-dimensional (3D) structures of protein-ligand complexes. The X-ray structures of complexes from the Protein Data Bank [1] are usually used for structure-based pharmacophore modeling. However, X-ray structures represent only a static view and can fail to describe the complexity of ligand–protein interactions. Protein-ligand complexes are inherently flexible species and their dynamic behavior greatly determines protein-ligand recognition. Molecular dynamics (MD) is a well-established approach for the simulation of the flexibility of large molecular systems and is widely used for the investigation of protein-ligand complexes’ dynamic behavior. MD simulations act as a rich source of information about studied systems, and thus can be used for drug design purposes. In particular, ensemble docking [2,3] employs individual snapshots of MD trajectory.

In several recent studies, researchers applied pharmacophore modeling for MD trajectory analysis. Choudhury et al. derived models from snapshots of a 40 ns trajectory and validated them on the external set of known active and inactive compounds to select the most reasonable pharmacophores [4]. They obtained only eight pharmacophores by selecting snapshots every 5 ns of the trajectory. Such amounts of pharmacophores are not only unrepresentative, but also this approach is applicable only if there are enough data on known active and inactive compounds for model validation and selection (because a priori is impossible to estimate the usefulness of models for virtual screening). Other researchers have clustered MD trajectories to select representative pharmacophore models [5,6] which reduced computational complexity due to fewer models. However, such approaches depend on a chosen clustering algorithm and its tuning parameters and can overlook some less populated states, which might be important for ligand-receptor recognition. Each of these approaches also requires datasets of known compounds to validate and select the most appropriate and accurate models.

Recently, Wieder et al. proposed the “common hits approach” (CHA) which requires no information about known ligands to validate and select predictive pharmacophore models [7]. They proposed the use of all representative pharmacophore models retrieved from a single MD trajectory of a protein-ligand complex to rank compounds according to the number of matched models. They demonstrated high performance of the CHA on a number of protein-ligand complexes. Nevertheless, the proposed selection procedure of representative pharmacophore models in that study has some weaknesses. The authors retrieved 20,000 MD trajectory snapshots and the corresponding number of pharmacophore models. To select representative pharmacophore models they grouped all models according to the number and types of pharmacophore features. The energy of ligand conformations corresponding to each pharmacophore model was calculated with the Merck Molecular Force Field (MMFF). A conformer with median energy was identified within each group and the corresponding pharmacophore model was selected as representative. The spatial arrangement of features was ignored because pharmacophore models were grouped only by the type and the number of pharmacophore features. Therefore, dissimilar pharmacophores with substantially different geometry but the same set of features can get to the same group, which will not correspond to a single representative model.

Nevertheless, there is a need to develop a stable approach capable of selecting representative pharmacophore models with a minimal number of tuning parameters. In this study, we used previously developed 3D pharmacophore hashes [8] which were able to identify identical pharmacophore models within a given binning step. A 3D pharmacophore hash is a unique identifier of a pharmacophore that takes into account distances between features and their spatial arrangement, including stereoconfiguration. A binning step, the only sensitive tuning parameter, was used for discretization of interfeature distances to enable fuzzy matching of pharmacophores by calculated hashes. The removal of pharmacophores with duplicated hashes reduced the whole set of pharmacophore models retrieved from the MD trajectory to a subset of representative ones, further used for virtual screening.

We also proposed a new approach of compound ranking, called the “conformers coverage approach” (CCA). Similar to the common hits approach, it uses all representative pharmacophore models, and therefore does not require validation and selection of individual models based on sets of known active and inactive compounds. Supposedly, if a greater number of existing compound conformers can fit protein conformational states, the more favorable binding would be observed as flexibility of a compound that better corresponds to flexibility of a protein (thus the ligand would lose fewer degrees of freedom upon a binding event and less binding entropy decrease may be observed). In the case when multiple complexes of a protein with different ligands are available, a consensus ranking can be performed by averaging CCA scores across different complexes. We also demonstrated the influence of pharmacophore model complexity represented by the number of features on virtual screening performance. The proposed approach to retrieve pharmacophore models from MD trajectory and virtual screening was implemented in open-source software available on GitHub (https://github.com/ci-lab-cz/pharmd).

## 2. Materials and Methods

### 2.1. Protein Target and Compound Dataset

**Figure 1:**
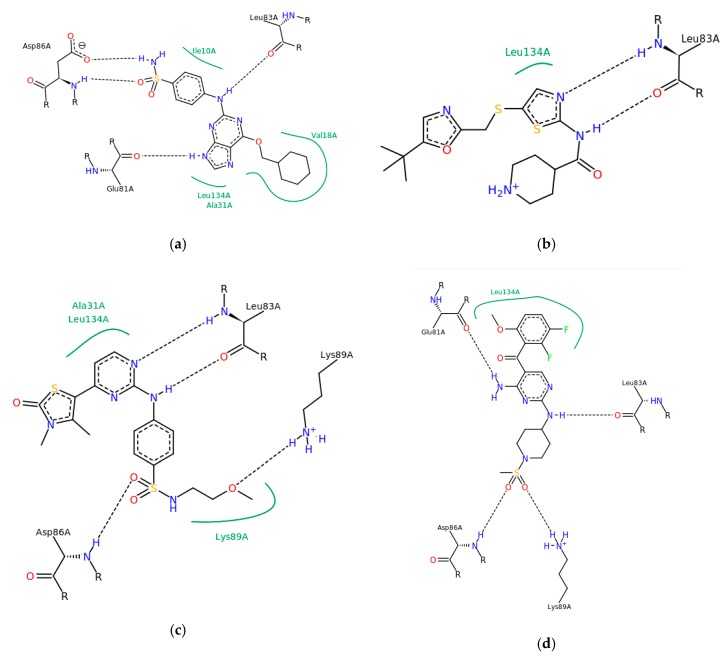
Protein–ligand interaction charts of four selected cyclin-dependent kinase 2 (CDK2) complexes. (**a**) IC_50_ = 5–8.1 nM [11,12,13] 2C6O; (**b**) IC_50_ = 38–46 nM [14,15] 5D1J; (**c**) K_i_ = 0.11 nM [16] 2XMY; (**d**) K_i_ = 3 nM [17] 2FVD.

We chose cyclin-dependent kinase 2 (CDK2) as a protein target due to the abundance of X-ray structures of this protein in complexes with small molecules. Among the available PDB X-ray complexes, we selected four with high affinity inhibitors (2C6O, 2FVD, 2XMY, and 5D1J) (Figure 1). The corresponding dataset of known inhibitors and decoys from the DUD-E dataset [9] was used for the validation of developed pharmacophore models. After a thorough check, all duplicates were removed from the validation set. In addition, the ligands presented in the selected four complexes were removed from the DUD-E dataset to avoid overestimation of model performance. The final dataset contained 473 active compounds and 27,853 decoys. For compounds with an undefined configuration of stereocenters or double bonds, all possible stereoisomers were enumerated. For each stereoisomer of a compound, up to 100 conformers were generated, within the energy gap 100 kcal/mol from the lowest energy conformer, using the MMFF force field implemented in RDKit [10]. Such a large energy gap was deliberately chosen to cover larger conformational space because large and flexible compounds with polar or charged groups can form bent conformations, where oppositely charged groups are close together. In addition, we considered conformers with a root-mean-square distance less than 0.5 Å as duplicates and removed them.

### 2.2. Molecular Dynamic Simulations

All molecular dynamics simulations were done using GROMACS 2016 with GPU support [18,19]. First, protein and ligand topologies were prepared. In the original 2XMY structure from PDB, two possible ligand structures are overlapped. They have extremely low RMSD, therefore, we arbitrarily chose structure A. For protein topology generation, we used the Amber99SB-ILDN force field [20]. The ligand topologies were prepared with an Antechamber 17.3 [21,22] using GAFF2 force field parameters and checked using parmchk utility and manually.

Each protein-ligand system was placed in a dodecahedron water cell with a minimal distance to the cell wall of 1 Å. The TIP3P [23] model was used for water description. The maximum number of steps for energy minimization was 50,000, but for all four complexes, the steepest descent converged at approximately 1000 steps. After energy minimization, each system was NVT and NPT equilibrated (100 ps per each equilibration) following guidelines published by Justin Lemkul [24]. Then, equilibrated protein-ligand complexes were simulated under NPT ensemble with a V-rescale thermostat and a Parrinello-Rahman barostat at 310 K for 50 ns with 2 fs time step. The temperature (during NVT equilibration) and density (during NPT equilibration) were carefully monitored and found acceptably stable. The simulations’ convergence was analyzed using RMSD and gyration radius plots, as well as by temperature and density as additional parameters (see Supplementary Materials).

### 2.3. Pharmacophore Model Retrieval

Individual snapshots of each 20 ps of MD trajectory of a protein-ligand complex were extracted to PDB files using MDTraj library [25]. A total of 2500 snapshots were retrieved from the MD trajectory of each complex. Water molecules were removed because we were interested in the identification of only direct ligand–protein interactions, and also this significantly sped up pharmacophore recognition. From each snapshot, we retrieved a pharmacophore model by the identification of hydrogen bonds, hydrophobic and aromatic interaction centers between protein and ligand, using PLIP library [26]; electrostatic interactions were identified as short contacts (less than 3.8 Å) between the side chains of charged amino acids (Glu, Asp, Lys, Arg, and His) and oppositely charged ligands. The assignment of pharmacophore features was refined to satisfy pharmacophore feature patterns implemented in pmapper [8] which was further used to derive 3D pharmacophore hashes from individual pharmacophore models. The binning step and tolerance for the calculation of 3D pharmacophore hashes were set to default 1 Å and 0, respectively. The former represents how models tolerate deviation of distances between features, and the later represents the tolerance to deviation of pharmacophore quadruplets from planarity to calculate stereoconfiguration of a pharmacophore [8]. In our previous study, we observed an extremely weak effect from changing the tolerance parameter, and therefore it was set to 0. The pharmacophore features (such as H-bond donors and acceptors) were undirected due to shortcomings of the current pmapper implementation. Because models with undirected features are less specific, this could lead to somewhat lower hit rates and enrichment. More details about the computing of 3D pharmacophore hashes can be obtained by referring to our previous publication [8].

### 2.4. Virtual Screening With Ensembles of MD-Based Pharmacophore Models

**Figure 2:**
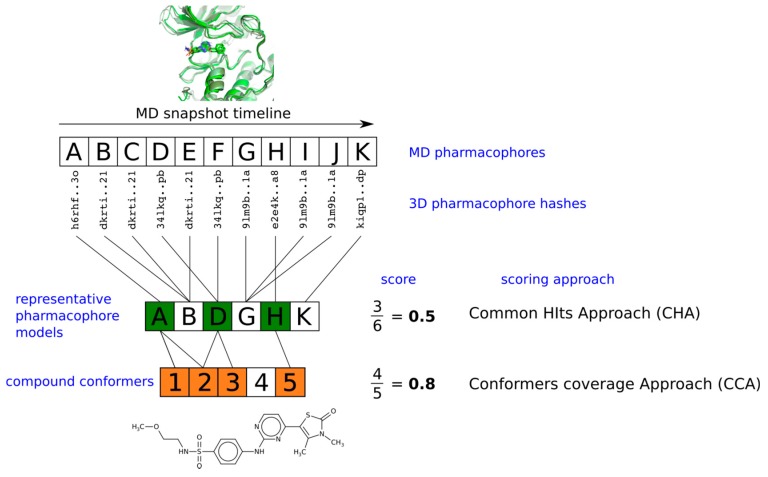
Compound scoring schemes based on the proposed conformers coverage approach and the previously developed common hits approach. Distinct representative pharmacophore models were selected among all molecular dynamics (MD) pharmacophores based on their three-dimensional (3D) pharmacophore hashes.

We reduced the number of considered pharmacophore models in each ensemble to representative ones by removing duplicates, i.e., pharmacophore models with identical hashes. Individual representative pharmacophore models were screened on the DUD-E dataset. Finally, compounds were ranked according to two strategies, common hits approach (CHA) and conformers coverage approach (CCA) (Figure 2). Within the CHA strategy proposed by Wieder at el., compounds were ranked according to the percentage of representative pharmacophore models matching at least one compound conformer, which was equivalent to the number of models matching the given compound from the original study [7]. It was suggested that active compounds should have a greater number of matched models. Within the proposed CCA strategy, compounds were ranked according to the percentage of conformers matching at least one representative pharmacophore model. We suggested that the compounds in which conformers fit more frequently to the pharmacophores observed within the MD simulations of a protein-ligand complex could have a more favorable binding due to less decrease in binding entropy. The more ligand conformers could fit the observed conformational states of a protein, the fewer degrees of freedom of a ligand would be lost upon a binding event.

To estimate screening performance, we calculated the precision, Equation (1), and the enrichment factor, Equation (2) which are the most important screening parameters, as the models should result in the lowest possible number of false positives and demonstrate enrichment over random selection. Enrichment was calculated at different percentages of selected compounds as follows: 0.25%, 0.5%, 1%, 2%, 5%, 10%, and 100%. Basically, we selected the specified percentage of compounds and all compounds having a score identical to the last compound in the list, and therefore the actual number of compounds could be greater than the given percentage. In addition, if the compounds retrieved by pharmacophore models was fewer than the given percentage we used only the retrieved compounds to calculate the statistics because the remaining compounds could not be ranked reasonably. All enumerated stereoisomers of a compound were treated as a single compound during virtual screening.

precision = TP/(TP + FP)(1)

enrichment factor = precision/baseline precision(2)

where TP is a number of true hits retrieved by a model, FP is a number of decoys retrieved by a model, baseline precision is calculated according to Equation (1) where all hits were considered as true positives and all decoys as false positives. The baseline precision for the DUD-E dataset was 0.0167.

## 3. Results and Discussion

**Figure 3:**
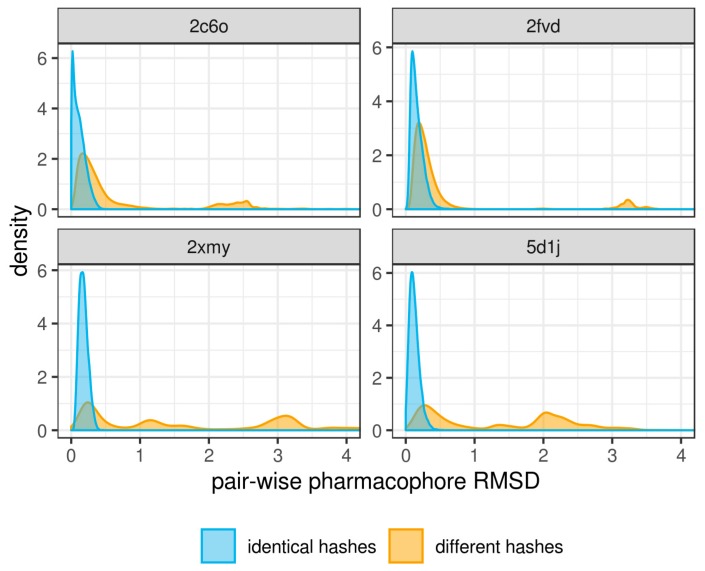
Gaussian kernel density of distribution of root-mean-square deviation values for the best fit between pairs of pharmacophores with identical and different hashes.

A total of 2500 frames were extracted from each MD trajectory of four complexes and the corresponding number of structure-based pharmacophore models was derived. Three-dimensional pharmacophore hashes were calculated for each pharmacophore to identify highly similar ones. By design, the pharmacophores with identical hashes should have a root-mean-square distance (RMSD) within the chosen binning step. In order to verify this, we aligned pairs of pharmacophore models with identical sets of features and calculated best-fit RMSD values. As expected, pharmacophores having identical hashes have a distribution of RMSD values from 0 to 0.93 Å across all four protein targets, whereas RMSD values for pairs of pharmacophores having different hashes were distributed in a wider range, from 0.01 to 4.96 Å (Figure 3). This indicates an important feature, i.e., identical 3D pharmacophore hashes always correspond to similar pharmacophores, however, similar pharmacophores do not always have identical hashes. This means that by removing pharmacophores with identical hashes we achieved the main purpose of reducing the number of pharmacophores, although keeping some redundancy among remaining representative pharmacophores.

**Figure 4:**
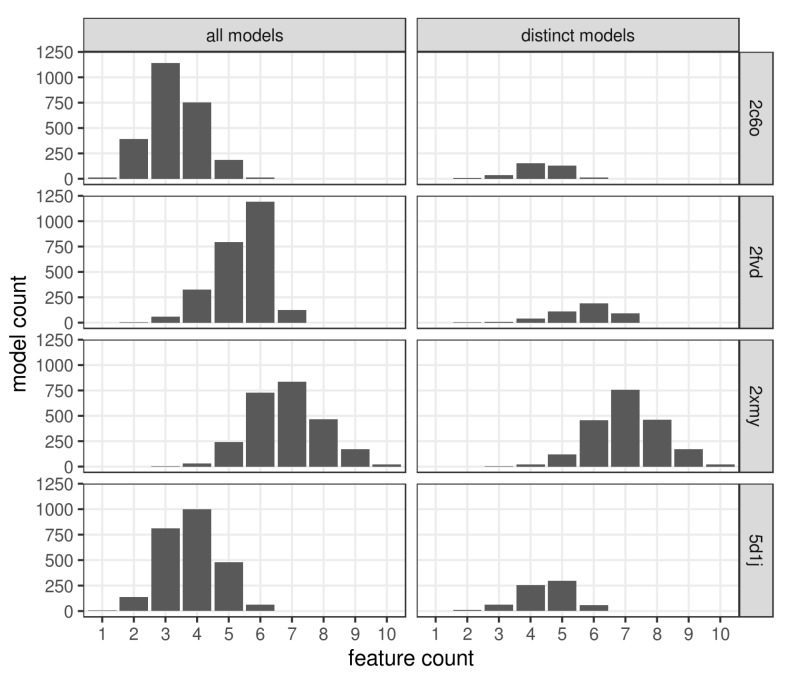
Distribution of all pharmacophores and pharmacophores having distinct 3D pharmacophore hashes according to their feature count.

Elimination of pharmacophores with duplicated hashes substantially reduced the number of pharmacophores for 2C6O, 2FVD, and 5D1J targets to 13.5%, 17.6%, and 27.3%, respectively. The pharmacophores retrieved for 2XMY target were the most diverse and the number of distinct pharmacophore hashes was high, 80.3% (Figure 4). This can be explained by higher flexibility of a 2XMY ligand and more complex pharmacophore models for 2XMY with a greater number of features than pharmacophores for other complexes.

We investigated the issue of how well the generated conformers of cocrystallized ligands reproduce binding modes represented by corresponding MD pharmacophores. Conformers of four cocrystallized ligands were generated using the same setup as for the DUD-E dataset. The most complex pharmacophores matched by 2C6O ligand were 16 four-feature pharmacophores. However, in general the complexity of corresponding MD pharmacophores was relatively low with only a few six-feature pharmacophores available (Figure 4). The 2FVD ligand matched five seven-feature pharmacophores which were the most complex among the available MD pharmacophores. It also matched 11 six-feature and 40 five-feature pharmacophores. The 2XMY ligand could match three six-feature and four five-feature pharmacophores, whereas the most complex MD pharmacophores contained 10 features. This can be explained by the high flexibility of a ligand that makes it difficult to find a conformer that perfectly matches such complex pharmacophores. The 5D1J ligand matched one five-feature and 13 four-feature pharmacophores, whereas the most complex MD pharmacophores had six features. These results indicate that the chosen setup of conformer generation can reproduce binding modes of cocrystallized ligands quite well.

To create baseline models, we retrieved pharmacophore models from initial PDB complexes using the same procedure. Only a four-feature PDB pharmacophore identified for 5D1J target had a hash identical to one of those retrieved from the MD simulations. The pharmacophore models for the remaining three complexes had a higher number of features relative to pharmacophores observed in the MD simulations (Figure 4). For the 2FVD complex, the PDB pharmacophore contained seven features, the number of representative MD pharmacophores of the same complexity was only 20%, and more complex pharmacophores were not observed. The 2XMY PDB pharmacophore consisted of nine features whereas only 9.7% of representative MD pharmacophores had the same or higher complexity. The PDB pharmacophore from the 2C6O complex had six features and there were just 15 representative MD pharmacophores of the same complexity (4.4%). On the one hand, due to a large number of features, the PDB pharmacophores extracted from the 2C6O, 2FVD, and 2XMY complexes were too specific and failed to retrieve any hits from the DUD-E dataset. On the other hand, the four-feature 5D1J PDB pharmacophore model was too loose and retrieved 10 hits and 818 non-hits that resulted in a poor enrichment equal to 0.72. The observation that PDB pharmacophore models can result in poor performance agrees with previous studies of other authors [4,7].

One expects that more complex pharmacophore models are more specific, which results in fewer retrieved hits and improves the chances of finding true hits. To estimate how the complexity of selected models affects virtual screening performance, we calculated enrichment based on all hits retrieved by any representative MD pharmacophores having at least a specified number of features (corresponds to EF_100%_). As expected, virtual screening based on all models with very simple pharmacophores having one, two or three features resulted in the lowest performance in all four cases. As the minimal complexity increased from a four-feature to a seven-feature model, enrichment improved and the number of retrieved hits significantly decreased (Table 1).

**Figure 5:**
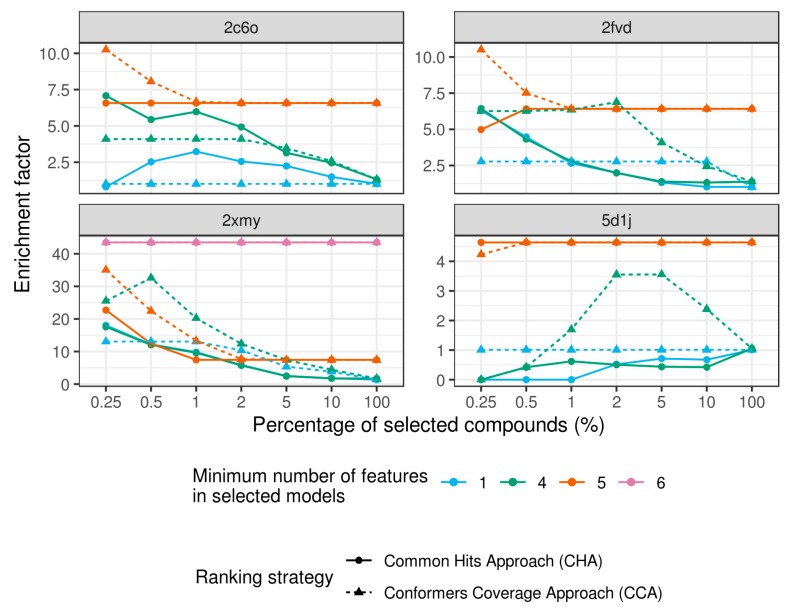
Enrichment factor for two ranking strategies at different complexity of selected models.

Due to the poor performance of the simple models, we used ensembles of models having at least four pharmacophore features while comparing two ranking strategies, CHA and CCA. In almost all cases, CCA demonstrated higher early enrichment factors than CHA (Figure 5). For example, for ensembles consisting of at least five-feature models, enrichment at 0.25% was 6.27 and 10.25 (2C6O), 4.98 and 10.5 (2FVD), 22.7 and 35.0 (2XMY), and 4.64 and 4.23 (5D1J) for CHA and CCA, respectively. A similar trend is observed for other percentages of selected compounds and model ensembles (Figure 5).

The ensembles of pharmacophore models consisting of a greater number of distinct complex models, such as in the case of 2XMY complex, result in better virtual screening performance. Early enrichment calculated within CCA at 0.25% and 0.5% was 43.5 and 35.0 for ensembles consisting of at least five- and six-feature models, respectively. This may suggest that complexes of ligands with a greater number of interactions are more preferable for virtual screening, if available.

We expected that using undirected features for H-bond donors and acceptors, as well as for aromatic features, would reduce virtual screening performance. Models with undirected features were less specific, and thus could retrieve more false positives. We did not investigate this issue explicitly because directed features were not implemented in the pmapper software. However, rather high early enrichments were achieved for many complexes, with up to 43.5 EF_0.25%_ for 2XMY. This indicates that directed features may sometimes be inessential. The only exception was the 5FVD complex in which we achieved only moderate enrichment for the ensemble consisting of at least five-feature models, EF_0.25%_ was 4.64. But this also may result from a specific binding mode of a ligand in the 5D1J complex not matched by other active compounds from the DUD-E dataset.

**Figure 6:**
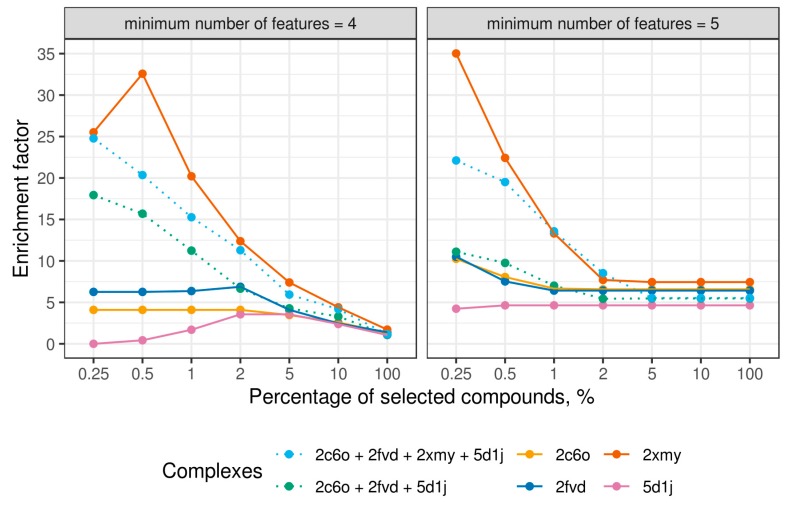
Enrichment factors for single pharmacophore ensembles and for consensus predictions made by averaging the scores of single compounds calculated for individual model ensembles within the conformers coverage approach.

In the case of several available X-ray structures, it is possible to combine predictions to improve screening accuracy. Compounds were ranked in descending order according to their average CCA scores calculated for different protein targets. We used only pharmacophore ensembles including models with at least four and five features because simpler models resulted in poor performance. More complex models were unavailable for all studied complexes. The consensus of four complexes demonstrated good performance in both cases with EF_0.25%_ being 24.8 and 22.1 (Figure 6). However, such high performance was mainly determined by high performance of the ensemble of pharmacophore models extracted from the MD trajectory of the 2XMY complex and the consensus ranking based on four complexes did not outperform the one for the 2XMY complex. Therefore, we evaluated consensus performance based on the average CCA scores among only three model ensembles with poorer performance (2C6O, 2FVD, and 5D1J). A substantial improvement was observed for the models having at least four features. The enrichment factor at 0.25% reached 17.9 for a consensus ranking, whereas it was 4.09, 6.26, and 0 for individual model ensembles of 2C6O, 2FVD, and 5D1J complexes, respectively. The improvement of consensus ranking, based on the output of ensembles comprising at least five-feature models, was less apparent as compared with individual model ensembles. The EF_0.25%_ was 11.1 for consensus ranking and 10.2 for 2C6O, 10.5 for 2FVD, and 4.2 for 5D1J. These results encourage the application of consensus ranking whenever possible because it decreases bias introduced by individual model ensembles and gives more robust output.

## 4. Conclusions

In this study, we demonstrated the advantages of “dynamic pharmacophores”, i.e., sets of pharmacophores extracted from snapshots of molecular dynamics trajectories, for virtual screening of biologically active compounds. The previously developed 3D pharmacophore hashes were successfully applied to identify identical pharmacophores and reduce the number of retrieved MD pharmacophores to representative ones. This approach omits complex calculations such as pharmacophore models clustering and selection of representative models. Since it linearly scales with a number of pharmacophore models, it can be freely applied to any number of snapshots. The 3D pharmacophore hash generation requires only one tuning parameter, i.e., binning step, which determines the fuzziness of hashes obtained. As we demonstrated, all pharmacophores with identical hashes had a pairwise root-mean-square distance less than the chosen binning step 1 Å.

We also proposed a new ranking approach, conformers coverage approach, based on the percentage of compound conformers matching representative pharmacophores from an ensemble of MD pharmacophores of an individual protein-ligand complex. Apparently, compounds with a high percentage of fitted conformers could lose fewer degrees of freedom upon binding and, as a consequence, binding entropy could be more favorable. We demonstrated that conformers coverage approach outperforms the previously proposed common hits approach on four selected protein-ligand complexes that supported the validity of the proposed approach. More rigorous validation on a larger number of complexes would be desirable.

As we observed, the usage of more complex pharmacophores with more features results in a higher performance of virtual screening. Models with three or fewer features are not recommended for virtual screening due to poor performance. As expected, there is a trade-off between the accuracy of predictions and the number of retrieved hits. Models of higher complexity result in higher enrichment values and less retrieved hits. Therefore, one should choose a model complexity depending on the particular goals of a study, but models should have at least four features and more.

Developed tools for the extraction of snapshots from a MD trajectory, assignment of pharmacophore features based on protein-ligand complex geometry, calculation of pharmacophore hashes, and virtual screening of compounds are freely available on GitHub (https://github.com/ci-lab-cz/pharmd).

## Figures and Tables

**Table 1:** The overall number of compounds retrieved from the DUD-E dataset by representative MD pharmacophore models of different minimum complexity.

PDB	Minimum Number of Pharmacophore Features in Models	Number of Representative Models	Number of Retrieved Compounds	TP/FP	EF_100%_^1^
2C6O	1	338	27,884 (98.6%)	471/27,413	1.01
	4	295	8109 (28.7%)	178/7931	1.31
	5	143	291 (1.03%)	32/259	6.58
2FVD	1	440	25262 (89.3%)	430/24,832	1.02
	4	431	7745 (27.4%)	180/7565	1.39
	5	390	205 (0.73%)	22/183	6.42
	6	282	2 (0.007%)	2/0	59.79
2XMY	1	2009	14,877 (52.6%)	337/14,540	1.35
	4	2008	10,470 (37.0%)	300/10,170	1.71
	5	1988	707 (2.5%)	88/619	7.44
	6	1868	33 (0.117%)	24/9	43.48
	7	1411	1 (0.004%)	1/0	59.79
5D1J	1	683	27,884 (98.6%)	471/27,413	1.01
	4	609	15,312 (54.1%)	270/15,042	1.05
	5	356	116 (0.41%)	9/107	4.64

^1^ Enrichment factor calculated for all retrieved hits.

## Supplementary Materials

Supplementary materials can be found at https://www.mdpi.com/1422-0067/20/23/5834/s1.
