# Supplementary material for: Virtual Screening Using Pharmacophore Models Retrieved from Molecular Dynamic Simulations
Source: Int J Mol Sci. 2019 Nov 20;20(23):5834. doi: 10.3390/ijms20235834 (PMC6929024; doi:10.3390/ijms20235834)
Supplement: Supplementary file 1 [file ijms-20-05834-s001.pdf]

Supplementary Materials:

Virtual Screening Using Pharmacophore Models Retrieved From Molecular Dynamic Simulations

Pavel Polishchuk [1,*], Alina Kutlushina [1], Dayana Bashirova [2], Olena Mokshyna [1] and Timur Madzhidov [2]

[1] Institute of Molecular and Translational Medicine, Faculty of Medicine and Dentistry, Palacky University and University Hospital in Olomouc, Hnevotinska 5, 77900 Olomouc, Czech Republic; alina.kutlushina@upol.cz (A.K.), olena.mokshyna@upol.cz (O.M.)

[2] A.M. Butlerov Institute of Chemistry, Kazan Federal University, Kremlyovskaya Str. 18, 420008 Kazan, Russia; dayana.bashirova@yandex.ru (D.B.), timur.madzhidov@kpfu.ru (T.M.)

* Correspondence: pavlo.polishchuk@upol.cz; Tel.: +420-585632298

PDB code	RMSD plot (compared with initial structure after least square fit to backbone)	Radius of gyration
2C6O	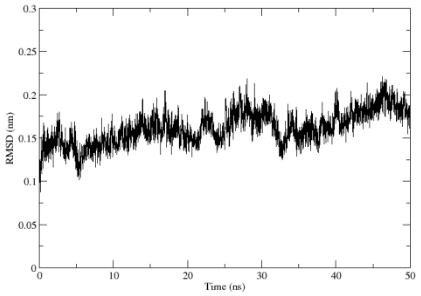	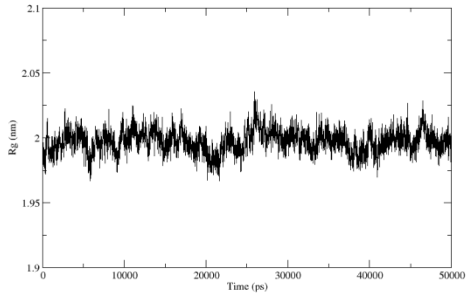
2FVD	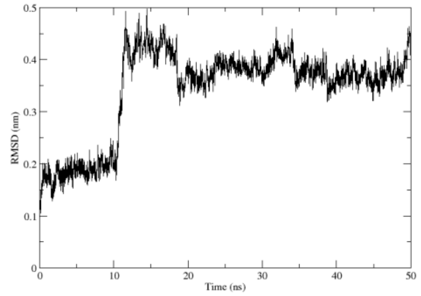	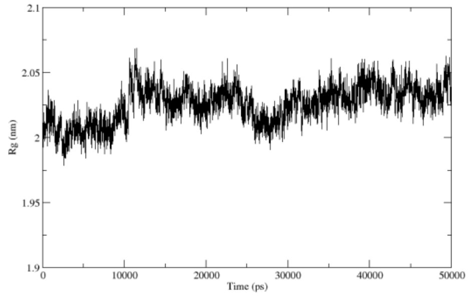
2XMY	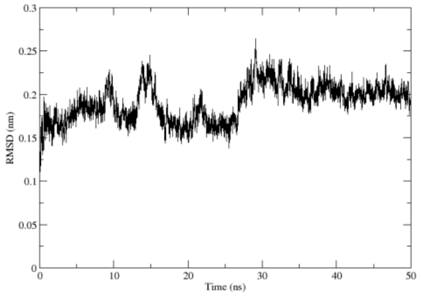	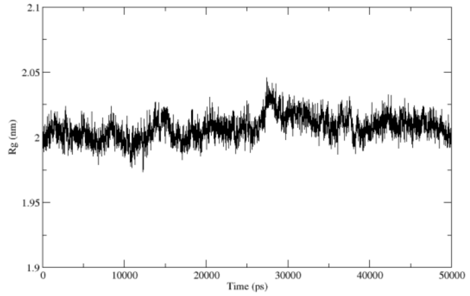
5D1J	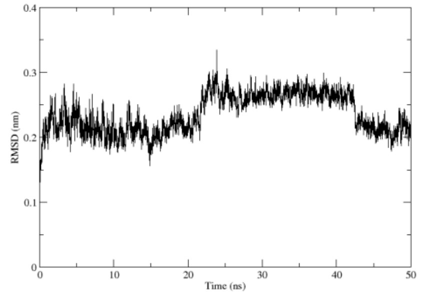	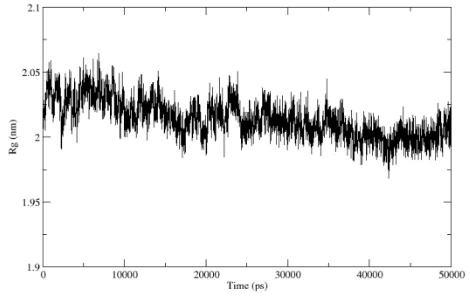

Figure S1. RMSD and radius of gyration plots from MD simulation of four studied complexes.

<table>
<tr><th>PDB code</th><th>Density</th><th>Temperature</th></tr>
<tr><td>2C6O</td><td>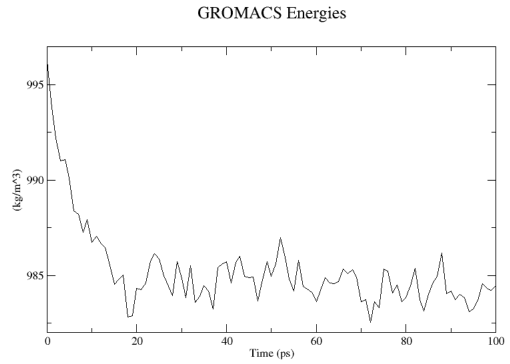
</td><td>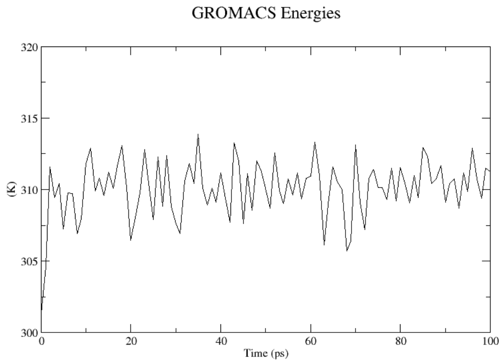
</td></tr>
<tr><td>2FVD</td><td>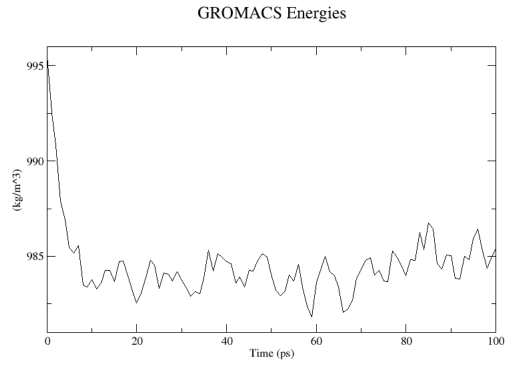
</td><td>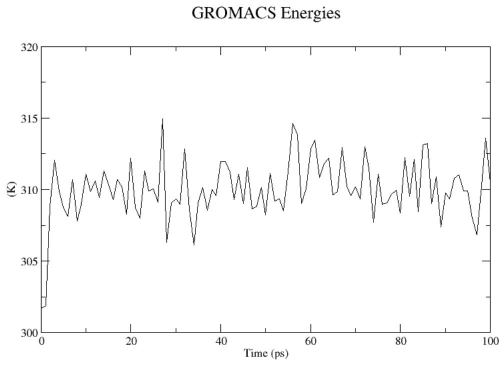
</td></tr>
<tr><td>2XMY</td><td>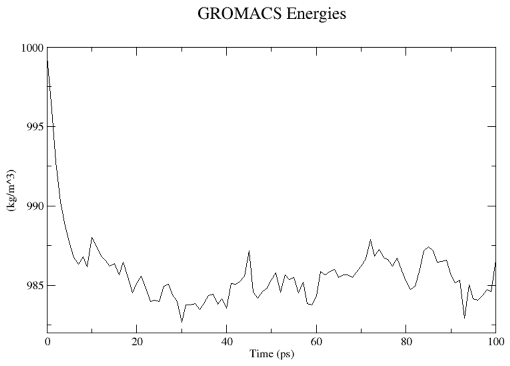
</td><td>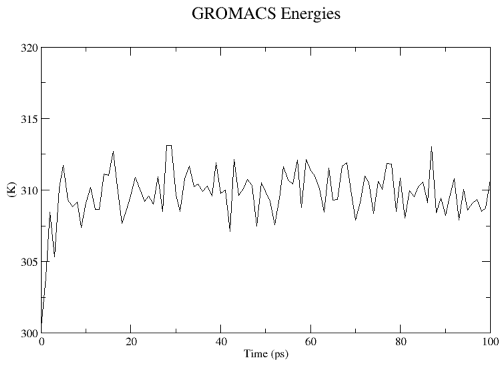
</td></tr>
<tr><td>5D1J</td><td>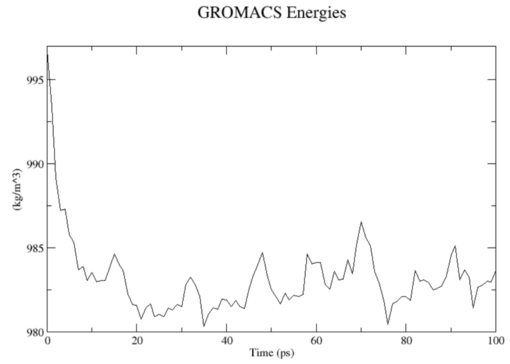
</td><td>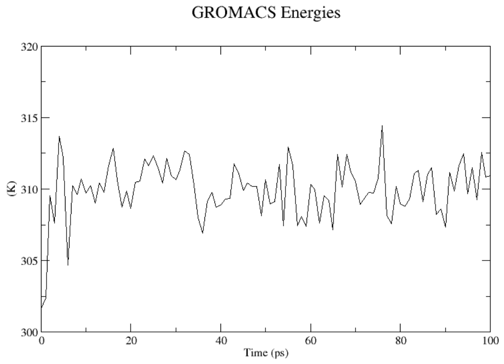
</td></tr>
</table>

Figure S2. Density and temperature plot during the equilibration stage of MD simulations of four studied complexes.

<table>
<tr><th>PDB code</th><th>Density</th><th>Temperature</th></tr>
<tr><td>2C6O</td><td>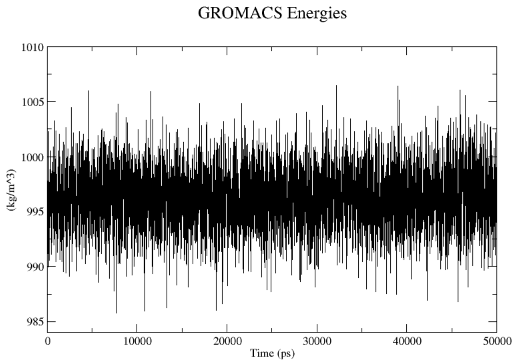
</td><td>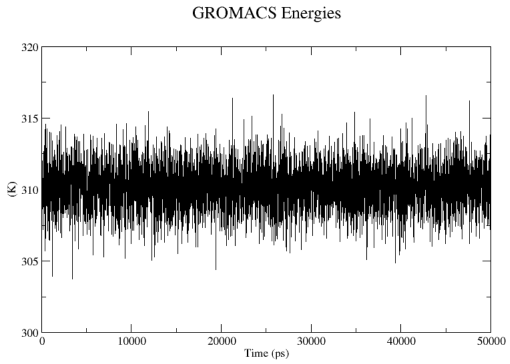
</td></tr>
<tr><td>2FVD</td><td>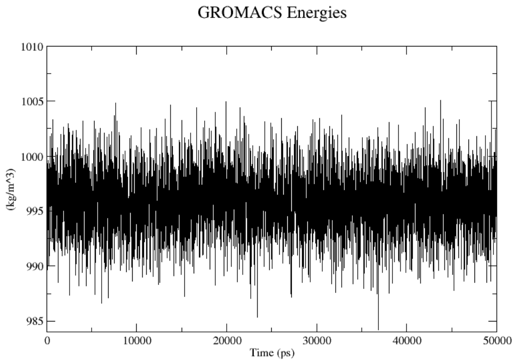
</td><td>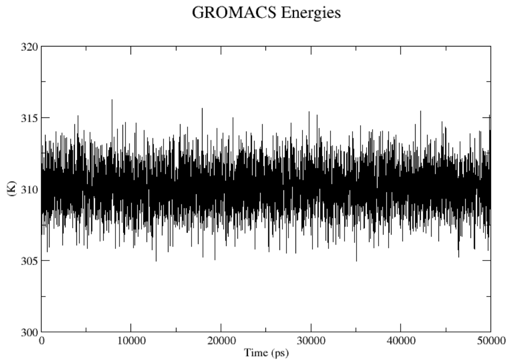
</td></tr>
<tr><td>2XMY</td><td>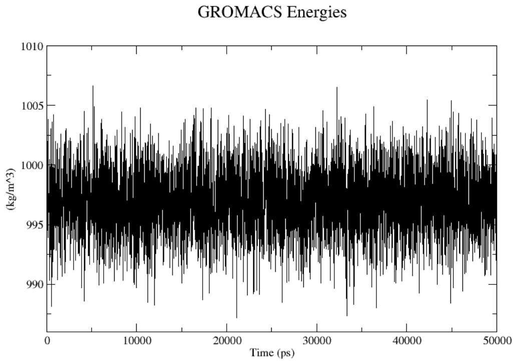
</td><td>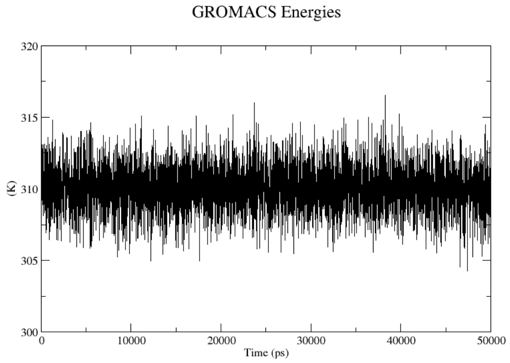
</td></tr>
<tr><td>5D1J</td><td>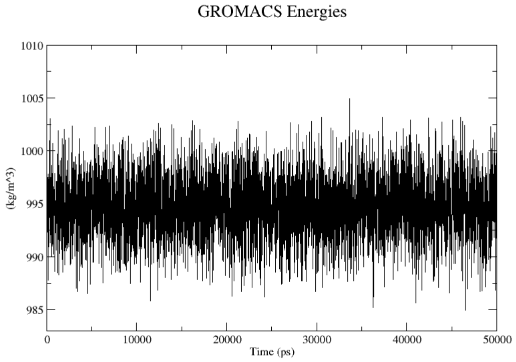
</td><td>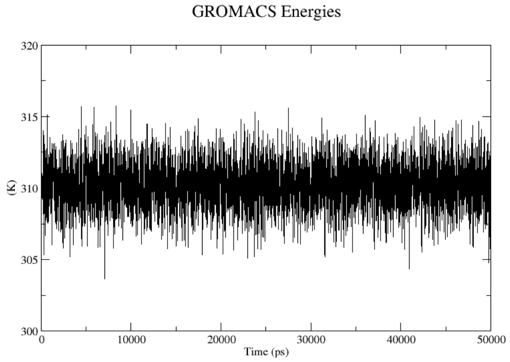
</td></tr>
</table>

Figure S3. Density and temperature plot during the production stage of MD simulations of four studied complexes.
